# Low Frequency Forced Oscillation Lung Function Test Can Distinguish Dynamic Tissue Non-linearity in COPD Patients

**DOI:** 10.3389/fphys.2019.01390

**Published:** 2019-11-19

**Authors:** Maria Ghita, Dana Copot, Mihaela Ghita, Eric Derom, Clara Ionescu

**Affiliations:** ^1^Dynamical Systems and Control Research Group, Ghent University, Ghent, Belgium; ^2^EEDT Core Lab on Decision and Control, Flanders Make Consortium, Ghent, Belgium; ^3^Department of Respiratory Diseases, Ghent University Hospital, Ghent, Belgium; ^4^Department of Automation, Technical University of Cluj-Napoca, Cluj-Napoca, Romania

**Keywords:** forced oscillation technique, respiratory impedance, COPD, viscoelastic properties, small airways, remodeling

## Abstract

This paper introduces the use of low frequencies forced oscillation technique (FOT) in the presence of breathing signal. The hypothesis tested is to evaluate the sensitivity of FOT to various degrees of obstruction in COPD patients. The measurements were performed in the frequency range 0–2 Hz. The use of FOT to evaluate respiratory impedance has been broadly recognized and its complementary use next to standardized method as spirometry and body plethysmography has been well-documented. Typical use of FOT uses frequencies between 4–32 Hz and above. However, interesting information at frequencies below 4 Hz is related to viscoelastic properties of parenchyma. Structural changes in COPD affect viscoelastic properties and we propose to investigate the use of FOT at low frequencies with a fourth generation fan-based FOT device. The generator non-linearity introduced by the device is separated from the linear approximation of the impedance before evaluating the results on patients. Three groups of COPD obstruction, GOLD II, III, and IV are evaluated. We found significant differences in mechanical parameters (tissue damping, tissue elasticity, hysteresivity) and increased degrees of non-linear dynamic contributions in the impedance data with increasing degree of obstruction (*p* < 0.01). The results obtained suggest that the non-linear index correlates better with degrees of heterogeneity linked to COPD GOLD stages, than the currently used hysteresivity index. The protocol and method may prove useful to improve current diagnosis percentages for various COPD phenotypes.

## 1. Introduction

Standardized, clinical practice requires specific maneuvers from the subject (e.g., spirometry), i.e., maximal inspiratory and expiratory effort are required. For example, in order to differentiate between patients with asthma and chronic obstructive disease (COPD) spirometry in combination with other techniques (e.g., broncho-dilation) is employed. Therefore, measurement and estimation of impedance by means of forced oscillation technique (FOT) have been widely investigated for several years to show its added value and complementarity (Kaczka and Dellaca, 2011; Oostveen et al., 2013; Kamada et al., 2017, 2018). This is a non-invasive procedure which does not require any special maneuvers from the patient and it requires minimal effort which makes this method an ideal lung function test, especially for the limit ages (children and elderly). FOT can be briefly described as an oscillatory air flow superimposed on the breathing of the patient (Ionescu, 2013). FOT has been broadly used for screening purposes, e.g., upper airway obstruction, small airway disease in COPD (Ionescu, 2013), respiratory mechanics in obstructive sleep apnea.

The non-invasive FOT is used to measure the respiratory mechanics at low frequencies (i.e., 0.1–2 Hz) in two groups of volunteers (i.e., adults and children). FOT is a non-standardized lung function test based on the action-reaction principle applied to the lungs during normal breathing in a non-invasive manner. The patient is advised to breath normally, i.e., no force maneuvers are required, a non-condition which enlarges significantly the applicability scope to marginal groups such as infants, children, and the elderly. Developed half century ago, FOT implies the generation of a signal *U*_*g*_(*t*) (sinusoidal, or combination hereof) within 0.3 kPa peak to peak amplitude. Signal generation has varied in the past decades from loudspeakers, mechanical pistons, to fan-based ventilators, depending on the envisaged range of frequencies and power to be applied to the patient's lungs (Olarte et al., 2015). For instance, loudspeaker based mechanisms are commonly used for frequencies well above the breathing frequency; 5–250 Hz, revealing properties of the proximal airways and useful for aerosol deposition studies. Mechanical actuators and ventilators works at lower frequencies (<5 Hz) enabling information on lung tissue properties such as viscoelasticity.

For the measurements performed in this paper, the device described in Olarte et al. (2015) has been employed. A more detailed description of the system can be found in Olarte et al. (2015). As mentioned above, when measuring at low frequencies interference between the breathing signal and excitation signal occurs. For this, a non-linear estimator has been employed to eliminate the disturbance signal (i.e., breathing) without losing the information about the respiratory response. However, the mathematical details have been described elsewhere (Ionescu et al., 2011a; Ionescu, 2013; Olarte et al., 2015; Copot et al., 2017a,b).

In the current study, we propose to evaluate low frequencies FOT in the presence of breathing signal and in a fourth generation custom-made lung function FOT device. The objective is to evaluate the sensitivity of FOT to various degrees of obstruction in COPD patients and to separate from the recorded information the linear and non-linear components of the respiratory impedance. To this purpose, three COPD groups are evaluated: GOLD II, GOLD III, and GOLD IV diagnosed patients. In this paper the hypothesis whether changes in tissue distribution in COPD will induce changes in the non-linear dynamic response has been investigated. A method and protocol is proposed to determine the amount of degree and correlate it to the GOLD classification of COPD.

The paper is structured as follows. The next section presents the biometric and spirometric details for the subjects and patients evaluated in this work. The measurement device and method for estimating respiratory impedance is also described. The third section illustrates the results, followed by a discussion section. A conclusion section summarizes the main ideas of this study.

## 2. Materials and Methods

### 2.1. Patients

In this paper a total number of 63 patients with COPD with an average age of 60 years old have been evaluated using the FOT device. The patients were coming for periodic lung evaluation at the pneumology department at Ghent University Hospital, Belgium. Biometric and spirometric parameters are shown in Table 1. Each patient signed the inform consent form and the protocol has been approved by the local ethical committee, registered under the number B670201526182.

**Table 1:** Biometric characteristics and baseline lung function data for the patients included in the study.

**–**	**GOLD II**	**GOLD III**	**GOLD IV**	**Baseline**
# Patients	21	22	20	20
Age (years)	60–71	65–73	70–74	33–36
Height (m)	1.68 –1.74	1.69–1.73	1.54–1.63	1.73–1.78
Weight (kg)	58–68	68–77	75–85	74–77
FEV1%pred	67–72	42–48	27–31	98–105

### 2.2. Measurement Device

**Figure 1:**
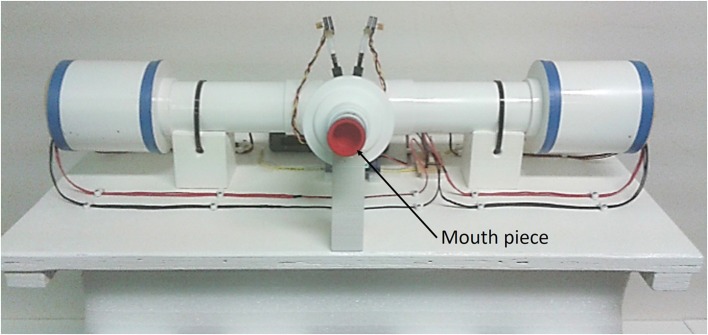
Photo of the device used for measurements. The patient is using the mouth piece to connect to the device and the flow is directed toward the patient while he breaths using straws. PWM stands for pulse wave modulated signal.

The measurement device presented in this paper is a fourth generation prototype of the device detailed in Ionescu et al. (2014) and Maes et al. (2014). The measurement device consist in two sets of three fans, one on each side of the main pipe as shown in Figure 1. The aim of these fans is to move the air into the tube on one side and to extract the air at the other side. A pneumotachograph and two pressure sensors deliver the flow and pressure measured at the mouth during tidal breathing. The pressure signal generated by the device is a multisine within the 0.05–2 Hz frequency interval. The device has been described in detail in Olarte et al. (2015) and Copot et al. (2017a) and includes compensation of deadspace effects (Tang et al., 2005) through closed loop control of fan speed.

### 2.3. Identification Method

The most identification approaches of non-linear dynamic systems is to have small amplitude variations and a good SNR, to avoid non-linear distortions. In this paper, we propose to make use of this information in a systematic analysis.

In general, for multi-harmonic periodic signals, the quadratic non-linear effect is present at combinations of *f*_*i*_ − *f*_*j*_, while the cubic non-linear effect is identified for the triple combination *f*_*i*_ − *f*_*j*_ − *f*_*k*_ (with *f*_*i*_, *f*_*j*_, and *f*_*k*_ the frequencies present in the excitation signal).

Consequently, exciting the system with periodic signals with only odd harmonics will prevent the even non-linear distortions. This allows discriminating between even and odd non-linear contributions, while providing a best linear approximation (BLA) of the system (Pintelon and Schoukens, 2012). This concept has been successfully applied to respiratory impedance in loudspeaker based device and a motor-driven piston based device (Ionescu et al., 2011a, 2014).

The influence of non-linear distortions on the frequency response of measurements are quantified by using identification methods given in Schoukens et al. (2002) and Pintelon and Schoukens (2012). A brief description of the essential steps is given hereafter.

(1) $$\begin{array}{l} u\left(t\right)=\frac{1}{\sqrt{N}}\overset{N}{\underset{k=1}{\sum }}A_{k}\sin\left(\omega_{k}t+\phi_{k}\right) \end{array}$$

with the phases ϕ_*k*_ random uniformly distributed in the interval [0; 2π], ω_*k*_ = 2π*kF*_*s*_ the excited frequencies of the multisine, with *F*_*s*_ the frequency resolution of the signal, *N* the number of excited harmonics and *A*_*k*_ the spectrum amplitude.

Consider an input signal defined as a random phase multisine with *A*_*k*_ the non-zero amplitude for odd *k* values, ω_0_ = 2π*f*_0_ and *f*_0_ = 0.1 Hz, ϕ_*k*_ the phase uniformly and independently distributed in the [0; 2π] interval and *N* the number of sinusoids. The best linear approximation (BLA) of a non-linear system can be viewed as a minimization of the mean squared error between the true output of the non-linear system and the output of an approximated linear model. The estimated BLA Ĝ_*BLA*_(*jω*_*k*_) of a wide class of non-linear systems, obtained using (1) can be written as:

(2) $$\begin{array}{l} {Ĝ}_{BLA}\left(j\omega_{k}\right)=G_{BLA}\left(j\omega_{k}\right)+G_{S}\left(j\omega_{k}\right)+N_{G}\left(j\omega_{k}\right) \end{array}$$

with *G*_*BLA*_(*jω*_*k*_) the true best linear approximation (BLA) of the non-linear system, *G*_*S*_(*jω*_*k*_) the zero mean stochastic non-linear contributions and *N*_*G*_(*jω*_*k*_) the measurement noise (Schoukens et al., 2002). The stochastic non-linear contributions *G*_*S*_(*jω*_*k*_) can be extracted by averaging from a manifold of experiments *M* containing different phase realizations in the excitation signal from (1).

**Figure 2:**
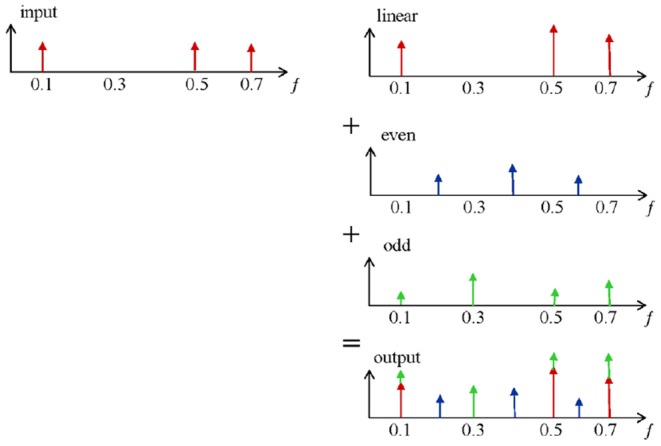
A schematic representation of the input-output contributions.

The basic principles for detecting non-linearities are shown in Figure 2. The output of a linear system,which is excited with a multi-frequency input signal,is given on the first row. Only amplitude variations on the excited (odd) frequencies are observed (red). However, when this input signal is applied on a non-linear system (e.g., the respiratory tissue), non-linear dynamics become visible and can be measured as additional detection lines (second and third row in blue and green). These distortions are in fact superimposed to the linear output signal and contribute to the signal measured at the output (last row). The resulting out-put signal contains extra information via phase differences. The non-linear contributions can be determined via the identification of the even and odd harmonics(blue and green). A detailed description of the method can be found in Schoukens et al. (2002) and Pintelon and Schoukens (2012). In order to reduce the non-linear effect over the FRF and distinguish between even and odd non-linearities, only odd frequencies are excited in the system. In this context, the even non-linearities do not contribute to *G*_*B*_(*jω*_*k*_). The presence and the level of even non-linearities can be detected at the even non-excited harmonics of the response spectrum signal. Additionally, detection of odd non-linear distortions can be identified by omitting some of the odd harmonics in the excitation (Pintelon and Schoukens, 2012).

A convenient excitation signal that allows controlling easily the excitation lines and maintains the advantages of periodic excitation is designed as in (1), where the even harmonics are not excited, and per group of three odd harmonics one is randomly omitted. To avoid that the random phase realizations affect the amplitude of the designed signal, a crest factor lower than 2.2 has been used in (1).

An in-depth systematic analysis of the device has been performed for detecting its non-linear contributions and its sensitivity to various respiratory tube calibers and lengths, along with a calibration validation, as given in Olarte et al. (2015). The respiratory impedance data from the patients presented in the current paper implies calibration and correction of non-linear contributions of the device.

**Figure 3:**
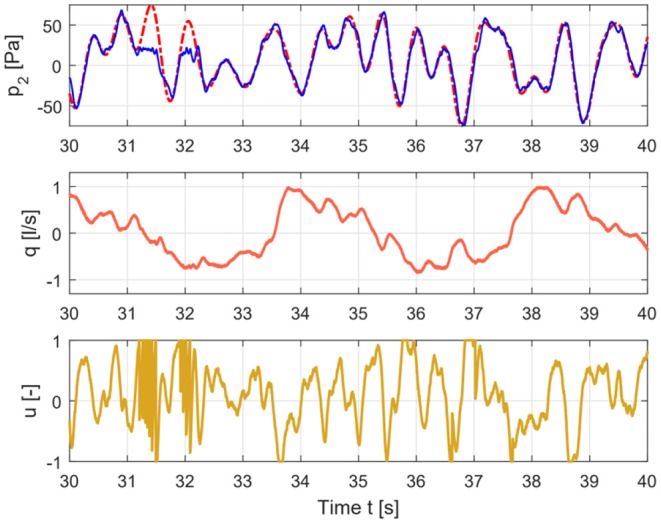
Time signals for healthy case depicting the input signal (lower graph), flow signal (middle graph), and pressure signal (upper graph).

In order to quantify these non-linear contributions, the following index has been introduced in Ionescu (2013) and it has been used in this paper to evaluate the degree of heterogeneity in patients with different degrees of COPD.

(3) $$\begin{array}{l} T=\frac{P_{even}+P_{odd}}{P_{exc}}\cdot \frac{U_{exc}}{U_{even}+U_{odd}} \end{array}$$

where *P* represents the pressure and *U* is the input signal (1). Each variable is the sum of the absolute values of all the contributions in pressure signal and input flow signal respectively, at the even non-excited frequencies, the odd non-excited harmonics and the excited odd harmonics as schematically depicted in Figure 3. Only the corrected output pressure has been taken into account when calculating (3), i.e., the linear contribution has been estimated and subtracted. The principle of detecting the non-linear contributions has been described elsewhere (Pintelon and Schoukens, 2012). A brief description of the method is given in the Appendix.

The index from (3) expresses a relative ratio of the contributions at the non-excited frequency points, with respect to the contributions at the excited frequency points. Furthermore, it gives a relative measure of the gain between contributions in the input and in the output of the system. Since this is a non-linear system whose output depends on the input, the choice for this relative measure is technically sound if one aims to extract the degree of non-linearity existent in lung tissue.

### 2.4. Estimating Impedance and Related Parameters

After the signals are recorded, they undergo a pre-conditioning step. The purpose is to eliminate the non-linear harmonics unavoidable by the interference with the low breathing frequency. For this, we employ the method proposed in Markovsky (2012) and Markovsky et al. (2014). This pre-conditioning leaves the data ready for estimation of respiratory impedance as real-imaginary parts as a function of frequency, with minimized effect from breathing dynamics. As the algorithm is not our contribution, is not listed here.

The subjects were asked to breath freely in normal sitting position for 30 s, i.e., about 5 periods of multisine excitation. The pressure (kPa) and flow (L) time based signals were sampled at 500 Hz frequency. The non-parametric estimation of the impedance was performed by employing the tools detailed in Daroczy and Hantos (1982), Oostveen et al. (2013), and Ionescu et al. (2014) based on spectral analysis techniques:

(4) $$\begin{array}{l} {Ẑ}_{BLA}\left(j\omega_{k}\right)=\frac{S_{PU}\left(j\omega_{k}\right)}{S_{QU}\left(j\omega_{k}\right)} \end{array}$$

where *S*_*PU*_, *S*_*QU*_ denotes the cross spectral density function between the output pressure measured at the mouth of the patient and the excitation signal, and the output flow and the excitation signal respectively, evaluated at the excited frequency lines ω_*k*_ (rad/s) corrected by the effect of non-linear contributions from the device. The result is a graphical evaluation of the respiratory properties and it is usually represented by its real and imaginary parts as a function of frequency.

To this data, non-linear least square identification algorithm can be applied to fit the parametric model:

(5) $$\begin{array}{l} Z_{CP}\left(j\omega_{k}\right)=R+I\left(j\omega_{k}\right)+\frac{1}{C{\left(j\omega_{k}\right)}^{\beta }} \end{array}$$

where *R* (kPa s/L) and *I* (kPa s^2^/L) represent the main airway resistance and inheritance, while the last term consists of a constant-phase element which can be quantified in term of imaginary and real part which will represent the tissue elastance and damping respectively, and are described by the following equations:

(6) $$\begin{array}{l} \begin{array}{c} G=\frac{1}{C\omega_{k}^{\beta }}\cos\left(\beta \pi /2\right)\\ H=\frac{1}{C\omega_{k}^{\beta }}\sin\left(\beta \pi /2\right) \end{array} \end{array}$$

The ratio between the elastance and damping represents tissue heterogeneity. When the changes in *R* are small then any changes in *G* (kPa s^(1−β)^/L) will depict changes in parenchyma or in very small airways (Ionescu et al., 2017). Significant changes in *H* (kPa s^(1−β)^/L) changes in the intrinsic mechanical properties of the parenchyma (Hantos et al., 1993; Thamrin et al., 2004).

This parametric model has been shown to reliably estimate airway and tissue properties (Ionescu, 2013). The accuracy of model parameters estimates by means of model fitting to experimental data indicates that the resistance and inheritance contain a high degree of uncertainty. The phase constancy parameter provides a reliable estimation of the peripheral tissue characteristics. The outcome of this study also indicates a dependency on frequency at low frequencies in the real part of impedance is consistently fitted by the constant-phase model from (5) when compared to other models from literature.

We have also previously showed that FOT measurements with both (4) and (5) are useful to characterize differences between healthy subjects and COPD patients (Ionescu et al., 2010a). We have shown in the past that the proposed models, method and device can be used to differentiate between healthy and disease subjects. However, we did not employ the non-linear index to evaluate the differences between the different degrees of COPD (i.e., COPD II, COPD III, and COPD IV). Hence, the aim of this study is to investigate whether or not the non-linear index can be used to distinguish between various degrees of obstruction in COPD. We have shown that the term in fractional order frequency arises naturally from the structure and mechanical properties of the lungs, as described in Bates et al. (1994, 2007); Suki et al. (1994), and Ionescu et al. (2010b, 2011b).

**Figure 4:**
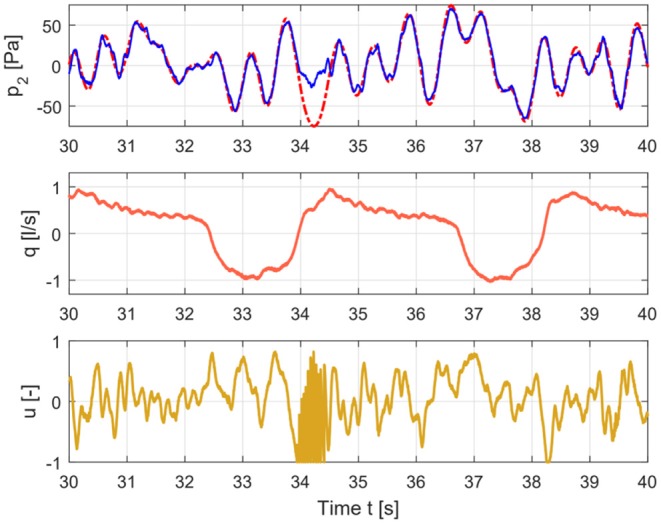
Time signals for COPD case depicting the input signal **(lower graph)**, flow signal **(middle graph)**, and pressure signal **(upper graph)**.

The measured pressure and flow and the input signal for healthy and COPD case are shown in Figures 3, 4.

**Figure 5:**
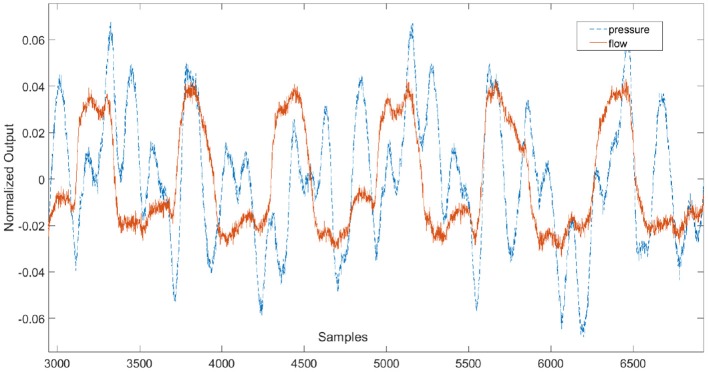
Example of pre-conditioned time signals used for the estimation of the respiratory impedance.

Figure 5 depicts a pre-conditioned pressure and flow signal used for the estimation of the respiratory impedance.

**Figure 6:**
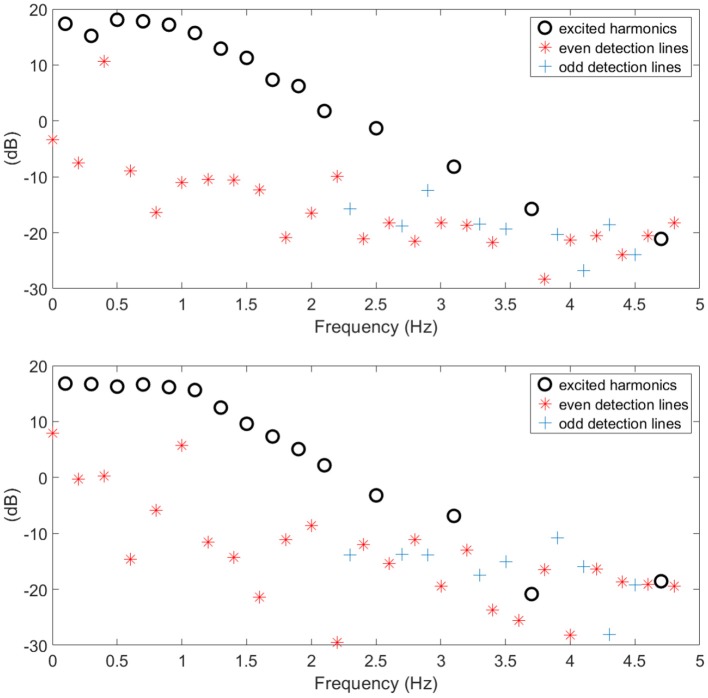
Example of healthy **(upper)** and COPD **(lower)** estimated BLA impedance modulus along with non-linear contributions used to calculate index T.

**Figure 7:**
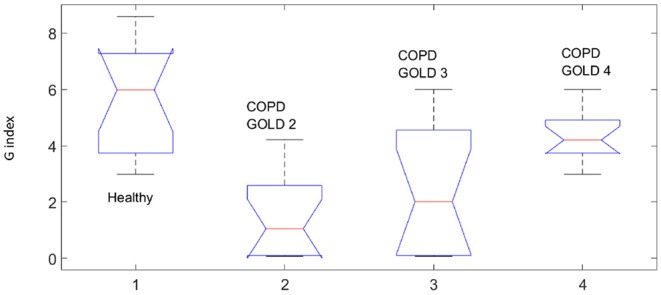
Evolution of the index G per group (*p* ≪ 0.001).

The BLA of the respiratory impedance modulus evaluated in healthy and in COPD GOLD3 are depicted in Figure 6 upper and lower, respectively. One may conclude the non-linear distortions tend to be more significant in patients with COPD than in healthy subjects as it can be seen in Figure 7. From clinical insight, this is indeed a valid conclusion, more details regarding the clinical interpretation can be found in Ionescu (2013) chapter 2. The respiratory system affected by the COPD disease changes its structure and this will also change the heterogeneous appearance of the tissues and will introduce non-linear effects such as: inflammation, clogged airways, viscoelasticity, etc. This paper presents a preliminary study on the further development of tools and methods for low-frequency measurement in a non-invasive manner. Although this evaluation is performed on a limited number of patients it indicates that measuring the non-linear contributions is beneficial to gather insight into evolution of respiratory diseases. Development of algorithms for canceling the interference with the breathing signal of the patient is motivated by the fact that the respiratory mechanics have inherent information on the viscoelastic properties of airways and tissue.

**Figure 8:**
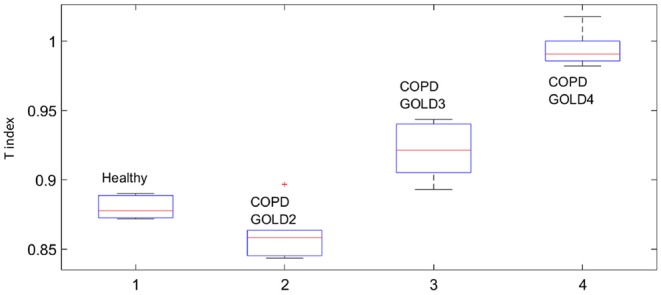
Evolution of the index T per group - some indicative difference exists (*p* ≪ 0.001).

This fact is also supported by the values obtained for the *T* index depicted in Figure 8 by means of Anova analysis plot (Matlab). More details about the non-linear index can be found in Copot et al. (2017a). Given that the number of subjects evaluated in each group is not very high one may conclude that at this stage a statistical measure cannot be performed, however a trend may be observed which corresponds to the expected result: heterogeneity in small airways will induce higher amount of non-linear effects in respiratory mechanics properties.

Statistical analysis was performed using the Anova plot in Matlab. With respect to airway resistance *R* and airway inheritance *I* these were not used to perform any further analysis since there was no normal distribution of the estimated parameters. The values estimated at each frequency point according to (6) and for all subjects in group are depicted as one column in the Anova plot, indicating that statistical difference exists, with *p* ≪ 0.001.

Our results suggest that the proposed non-linear dynamic index for the respiratory function *T* is better correlated to heterogeneity in the COPD GOLD stages than the broadly used heterogeneity index η.

The values estimated at each frequency point and for all subjects in group are depicted as one column in the Anova plot, indicating that statistical difference exists, with *p* ≪ 0.001.

## 3. Discussion

The presence of non-linear contributions in the respiratory system increased steadily with every group, indicating a more pronounced linear behavior in healthy lungs, depicted in Figure 8. This does not imply a perfect linear system, but rather a *less non-linear* one. Pathological changes in COPD affecting structure and viscoelastic properties introduce dynamical effects, which may move away from Newtonian rheological framework. In the remodeling framework associated with the evolution of COPD (Hogg et al., 2004; Bergeron and Boulet, 2006), thickening of the airway wall and accumulation of inflammatory exudates in the lumen will enhance turbulent airflow conditions which will enable significant non-linear contributions in the dynamical system under analysis (Ionescu et al., 2011a). In patients with GOLD III and GOLD IV, severe increase in lymphocytes and presence of lymphoid follicles will determine a drastic change in the energy balance distribution for enabling airflow in small airways (Williamson et al., 2011). This accounts for significantly higher degree of non-linearity in these groups than in GOLD II.

The degree heterogeneity in the small airways is related to the presence of non-linear contributions. In Bates and Allen (2006), a detailed description on the interpretation of the index with changes in elastance and resistance is given. With respect to η For η to increase by heterogeneity at low frequencies (i.e., f = 0.16 Hz), the resistive and elastic properties must have appropriate relative values. The regional increase/decrease in heterogeneity does not automatically lead to an increase in η (Bates and Allen, 2006). This specific aspect is reflected in COPD GOLD III data, where the hysteresivity factor has a high variability interval compared to the other groups. The relative high heterogeneity factor in healthy subjects may be due to the high variability present in the healthy respiratory system to allow high flexibility to changing environmental and operating conditions. This variability is also the backbone for adaptation and remodeling with disease. Our respiratory tree is rather robustly designed and heterogeneity in healthy does not have the same underlying principles as the structural changes due to disease.

Significant changes in *G* or *H* parameters indicate enhanced or altered secretions that cause dysfunction in small airways (Hantos et al., 1993). Changes in *H* will induce changes in tissue elastance. Our values of *H* were slightly lower than in Lorx et al. (2009) for COPD patients, but this may be due to the fact that Lorx and colleagues evaluate *H* at specific transrespiratory pressures. They indicated that at frequencies below 2 Hz, the elastance values in COPD may be similar or even smaller than those in healthy respiratory system, indicating the decreased elasticity resulting from emphysematous changes. This is in accordance with out results in *H* values for COPD GOLD III and GOLD IV.

It has been concluded in Lutchen and Gillis (1997) that lung resistance and lung elastance are extremely sensitive to mild inhomogeneous constriction in which a few highly constricted or nearly closed airway are present in peripheral zones. Strong dependencies with frequencies have been observed at breathing rates. This conclusion from their simulation studies can be well-verified with the results obtained for COPD GOLD II group, where markedly increased values for *E*_*ti*_ have been found. This indicated mild inhomogeneous constriction. By contrast, severe homogeneous constriction would not increase *E*_*ti*_ at the lower breathing frequencies, supported by our results for COPD GOLD III and GOLD IV groups. However, it seems that our proposed non-linear dynamic index *T* outperforms the relevance of η in degrees of heterogeneity assigned to various stages of COPD GOLD.

In Dailey and Ghadiali (2010), the very same power law formulation from (6) has been used to characterize microrheological properties of materials in the context of cell-injury during specific loads. This may be relevant for instance in analyzing lung injury during mechanical ventilation and preset positive end-expiratory pressures (Wheeler and Bernard, 2007). However, the frequency dependency allows a dynamic characterization of viscoelastic properties in lungs or in similar materials (e.g., polymers). In Freed and Einstein (2012), a model for lung parenchyma has been developed where stress-strain properties are characterized for lung lobes, similar to a pressure/volume curve model for inflation/deflation. We also showed previously that mechanical models of lungs preserving properties and structure imply the presence of a power law term in time domain, or equivalently a fractional order term in frequency as in (5) (Ionescu et al., 2010c; Ionescu, 2013). However, simple impedance models as (5) are more accessible and easier for evaluation in a clinical context than complex theoretical models which are useful for understanding underlying mechanisms of lung recruitment and may be useful when individualized treatment and follow up of COPD patients are envisaged (Derom et al., 2007). This individualized treatment could very well be paired with a wearable monitoring system for respiratory impedance as that presented in Ionescu and Copot (2017).

## 4. Conclusions

In this paper we have shown that forced oscillation technique is a complementary tool in evaluating tissue heterogeneity and dynamic non-linearity in respiratory impedance data. The proposed non-linear index provides valuable additional information to the classical constant-phase parameter model. The protocol enables separating linear from non-linear effects, whereas changes in tissue parameters from constant-phase model are highly sensitive to changes in peripheral airways. The non-linear dynamics index proved to be better correlated to various stages of COPD GOLD than the currently used index for histeresivity. There is no doubt that FOT, in combination with reliable measurements at breathing frequencies, can deliver information about small airways and thus help in accurate diagnosis of various COPD phenotypes.

## Ethics Statement

All subjects gave their written informed consent and the protocol was approved by the local ethical committee, registered under the number B670201526182.

## Author Contributions

All authors listed have made a substantial, direct and intellectual contribution to the work, and approved it for publication.

### Conflict of Interest

The authors declare that the research was conducted in the absence of any commercial or financial relationships that could be construed as a potential conflict of interest.
