# Supplementary material for: Low Frequency Forced Oscillation Lung Function Test Can Distinguish Dynamic Tissue Non-linearity in COPD Patients
Source: Front Physiol. 2019 Nov 19;10:1390. doi: 10.3389/fphys.2019.01390 (PMC6877497; doi:10.3389/fphys.2019.01390)
Supplement: Supplementary file 1 [file Appendix.pdf]

APPENDIX

The influence of nonlinear distortions on the frequency response function (FRF) measurements are determined by using a random phase multisine (Pintelon and Schoukens, 2012; Schoukens et al., 2002):

$$U(t) = \sum_{k=1}^{N} A_k \cos(k\omega_0 t + \phi_k) \tag{A.1}$$

as an input signal, with A_k the non-zero amplitude for odd k values, $\omega_0 = 2\pi f_0$ and $f_0 = 0.1$ Hz, ϕ_k the phase uniformly and independently distributed in the $[0; 2\pi]$ interval and N the number of sinusoids. The best linear approximation (BLA) of a nonlinear system can be viewed as a minimization of the mean squared error between the true output of the nonlinear system and the output of a linear model. The estimated BLA $\hat{G}_{BLA}(j\omega_k)$ of a wide class of nonlinear systems, obtained using a random phase multisine, can be written as:

$$\hat{G}_{BLA}(j\omega_k) = G_{BLA}(j\omega_k) + G_S(j\omega_k) + N_G(j\omega_k) \tag{A.2}$$

with $G_{BLA}(j\omega_k)$ the true best linear approximation (BLA) of the nonlinear system, $G_S(j\omega_k)$ the zero mean stochastic nonlinear contributions and $N_G(j\omega_k)$ the measurement noise (Pintelon and Schoukens, 2012). The stochastic nonlinear contributions $G_S(j\omega_k)$ can be extracted by averaging from a manifold of experiments M containing different phase realizations in the excitation signal from (A.1). The measurement noise $N_G(j\omega_k)$ can be minimized by measuring longer, thus by increasing N. The basic principles for detecting nonlinearities are shown in figure 2. The output of a linear system, which is excited with a multi-frequency input signal, is given on the first row. Only amplitude variations on the excited (odd) frequencies are observed (red). However, when this input signal is applied on a nonlinear system (e.g. the respiratory tissue), nonlinear dynamics become visible and can be measured as additional detection lines (second and third row in blue and green). These distortions are in fact superimposed to the linear output signal and contribute to the signal measured at the output (last row). The resulting output signal contains extra information via phase differences. The nonlinear contributions can be determined via the identification of the even and odd harmonics (blue and green). Figure A.1 depicts a schematic

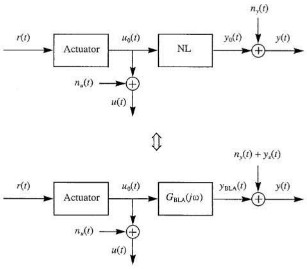

FIGURE A.1 | Input *u*(*t*) and output *y*(*t*) measurements with noise from a nonlinear system driven by an actuator with input signal *r*(*t*) (top), and its corresponding best linear approximation (under).

of the input and output measurements corrupted by noise and the corresponding BLA. In Pintelon and Schoukens (2012), a variance analysis procedure has been proposed that allows to detect and quantify the stochastic nonlinear distortions $G_S(j\omega_k)$ and the disturbing noise $N_G(j\omega_k)$. Two measurement methods can be applied:

- a robust method, which uses different random phase realizations of an odd multisine excitation to get information about the stochastic nonlinear distortions;
- a fast method, which uses only one realization of an odd random phase multisine with random harmonic grid.

The information about the stochastic nonlinear distortions is obtained via the detection lines (non-excited harmonics) in the output DFT spectrum. To compensate for spectral impurity of the input, a first order correction is applied to the output DFT spectrum. The use of an odd random phase multisine with a random harmonic grid provides also a classification of the nonlinear distortions in even and odd contributions in the corrected output DFT spectrum. Figure A.2 shows the principle of measuring the BLA using the robust method, i.e. by averaging between M realizations with P periods. If the input signal is known and uncorrupted with noise, one can obtain the non-parametric estimation of the BLA as $G_{BLA}(j\omega_k)$, the variance of the stochastic nonlinear distortions $var(G_S(j\omega_k))$ and the variance of the noise $\sigma^2_{GBLA,n}$. Given that $n_y(t)$ is a stochastic signal and $y_s(t)$ a periodic signal depending on the phase realization

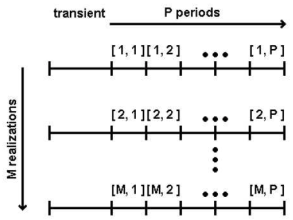

FIGURE A.2 | Measurement procedure for the robust method: *P* periods of length *N* measured from the steady state response with an odd multisine input. The experiment is then repeated *M* times, each time with a different odd random phase multisine realization.

of the input (reference) signal $r(t)$, the frequency response function of the mth realization and p period, $G^{[m,p]}(j\omega_k)$ can be written as:

$$G^{[m,p]}(j\omega_k) = G_{BLA}(j\omega_k) + \frac{Y_S^{[m]}(k)}{U_0^{[m]}(k)} + \frac{N_Y^{[m,p]}(k)}{U_0^{[m]}(k)} \tag{A.3}$$

Next, the BLA, variance of the nonlinear distortions and noise variance can be estimated as:

$$\hat{G}^{[m]}(j\omega_k) = \frac{1}{P}\sum_{p=1}^{P} G^{[m,p]}(j\omega_k) \tag{A.4}$$

$$\hat{G}_{BLA}(j\omega_k) = \frac{1}{M}\sum_{m=1}^{M} \hat{G}^{[m]}(j\omega_k) \tag{A.5}$$

$$\hat{\sigma}^2_{\hat{G}^{[m]}}(k) = \sum_{p=1}^{P} \frac{|G^{[m,p]}(j\omega_k) - \hat{G}^{[m]}(j\omega_k)|^2}{P(P-1)} \tag{A.6}$$

$$\hat{\sigma}^2_{\hat{G}_{BLA}}(k) = \sum_{m=1}^{M} \frac{|G^{[m]}(j\omega_k) - \hat{G}_{BLA}(j\omega_k)|^2}{M(M-1)} \tag{A.7}$$

$$\hat{\sigma}^2_{\hat{G}_{BLA,n}}(k) = \frac{1}{M^2}\sum_{m=1}^{M} \hat{\sigma}^2_{\hat{G}^{[m]}}(k) \tag{A.8}$$

$$var(G_S(j\omega_k)) \approx M(\hat{\sigma}^2_{\hat{G}_{BLA}}(k) - \hat{\sigma}^2_{\hat{G}_{BLA,n}}(k)) \tag{A.9}$$

Nomenclature:

- $U_0^{[m]}(k)$ - input of DFT spectra of mth FRF measurements;
- $Y^{[m,p]}(k)$ - output of DFT spectra of mth FRF measurements;
- $G_{BLA}(j\omega_k)$ - asymptotic best linear approximation;
- $\hat{G}^{[m]}(j\omega_k)$ - estimated spectrum of mth realizations;
- $G^{[m,p]}(j\omega_k)$ - frequency response function;
- P - number of periods;
- $\hat{G}^2_{BLA}(k)$ - estimation of best linear approximation;
- $\hat{\sigma}^2_{\hat{G}_{BLA}}(k)$ - estimation of the total variance averaged over M experiments;
- $\hat{\sigma}^2_{\hat{G}_{BLA,n}}(k)$ - the variance of noise averaged over M experiments;
- $var(G_S(j\omega_k))$ - variance of the stochastic non-linear distortions with respect to one multisine realization.

The total variance and noise variance averaged over the M experiments give an indication upon the reliability of the measured frequency response functions. The variance of the stochastic nonlinear distortion for each realization gives an indication of how much distortion is present in the system with each experiment. The fast method can be considered as a special case of the robust method, with $M = 1$. As such, the variance is expected to have higher values and less reliability for the fast method than in the robust method. For all the results presented in this paper, the robust method was employed with $M = 5$. Fast method needs odd detection lines and approximation in order to predict the odd distortion levels at the exited bins.
